# Vennter – An interactive analysis tool for WormBase interaction data using Venn diagrams

**DOI:** 10.17912/micropub.biology.000258

**Published:** 2020-05-25

**Authors:** Jaehyoung Cho, Sibyl Gao, Lincoln Stein, Paul W Sternberg

**Affiliations:** 1 Division of Biology and Biological Engineering, California Institute of Technology, Pasadena, CA 91125, USA; 2 Informatics and Bio-computing Platform, Ontario Institute for Cancer Research, Toronto, ON M5G0A3, Canada

**Figure 1:**
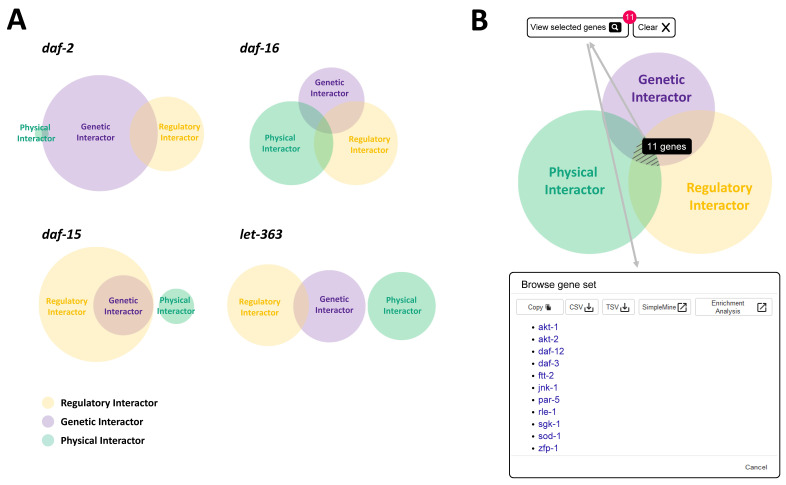
Vennter is an interactive Venn diagram tool for integrating different interaction data types as well as offering further analysis. (A) Examples of Vennter with different overlapping patterns among interaction data sets. (B) Vennter is an interactive tool providing hyperlinks to each gene page and batch analysis tools (SimpleMine and Gene-set Enrichment Analysis) for the genes selected from a specific area within the diagram (see hatched lines).

## Description

WormBase curates four different types of gene-to-gene interaction data: genetic, regulatory, physical, and predicted. These data are found in the Interactions widget in each gene page. Aside from the predicted interactions, the other three types are curated with direct experimental evidence from the literature. In WormBase, genetic interaction data is defined as a phenotypic deviation of double mutants (or any other genetic perturbations) from single mutant phenotypes and the control phenotype. Regulatory interactions are defined by how perturbation of one gene or gene product affects the expression of another gene or the localization of its gene product. Physical interactions represent molecular associations between genes and gene products from *C. elegans* (Grove *et al.* 2018). Each type of interaction data is essential to understanding certain aspects of the biological process mediated by the two interacting genes. However, integrating information from these three types of interaction data is critical to reading the biological context. Understanding the logical relations between different types of interaction data provides a vital clue on how to tackle a gene-to-gene interaction within this context. To achieve this goal, we introduce a new tool for analyzing these logical relations among the interaction data using a Venn diagram, named Vennter (**Venn** diagram for in**ter**action). Venn diagrams are very useful for displaying similarities and distinctions between different data sets of interest, especially when they are area-proportional to the amount of information presented.

Vennter consists of three circles, each representing one of the three different interaction data types. The size of any area within the interactive Venn diagram corresponds to the number of unique interactor genes pertaining to that region. Figure 1A shows an example of a Vennter diagram for selected genes (*daf-2*, *daf-15*, *daf-16*, and *let-363*) which play key roles in the Insulin/IGF-1 and TOR-dependent signaling pathway. These results exhibit distinct patterns of overlap among their interaction data. In some cases, the differences might reflect differences in prior scientific approaches to studying these genes as well as the availability of annotated interaction data in WormBase.

Vennter is fully interactive for analyzing interactor genes from different sets of interaction data. By clicking on any single or multiple area in combination, one can easily obtain all gene names corresponding to the selected area (represented by hashed lines) in Vennter. The number of selected genes is shown next to the ‘View selected genes’ button (see arrows). From there, one can open a popup window called ‘Browse gene set’ which contains a comprehensive list of interactor genes shown in alphabetic order. This list can be copied or downloaded in diverse formats, and for the user’s convenience, each gene name is linked to its unique WormBase gene page. Under the same ‘Browse gene set’ window, Vennter also offers other functions for further analysis of selected genes, such as direct links to the batch analysis tools ‘SimpleMine’ and ‘Gene-set Enrichment Analysis’ (Figure 1B) which can help to query the gene list more conveniently and efficiently.

To demonstrate yet another aspect of Vennter, we can look at the high-throughput physical protein-protein interaction data, which comprise long lists of genes. To date, WormBase has curated 86.9% of physical protein-protein interaction data from these high-throughput studies (Cho *et al.* 2018). However, identifying the functional relevance of such large amounts of interacting gene candidates can often be quite challenging to researchers. To improve this, Vennter provides more organized and relevant information to researchers by evaluating overlapping information between physical, genetic, and/or regulatory interactions, which enables researchers to more easily measure the confidence and biological relevance of their gene candidates of interest.

For the *C. elegans* research community, Vennter can help compare and integrate the different types of interaction data available. It also provides hyperlinks to other useful WormBase tools in order to analyze the gene interactor list in a more efficient and automated manner.

## Reagents

All the interactions data are available at the WormBase FTP site

(ftp://ftp.wormbase.org/pub/wormbase/releases/current-production-release/species/c_elegans/PRJNA13758/annotation/c_elegans.PRJNA13758.WS###.interactions.txt.gz, where WS### is the database version release, like “WS275”).

At the time of writing, the source code for Vennter is part of the WormBase website source code. The WS276.1 version of the source code is available at the GitHub depository (https://github.com/WormBase/website-public/releases/tag/WS276.1).
